# Analysis of the Thigh Aesthetic Profiles: One of Physical Ideal Body Proportions

**DOI:** 10.1007/s00266-024-03948-9

**Published:** 2024-03-25

**Authors:** Gkionoul Nteli Chatzioglou, Figen Govsa, Gokhan Gokmen, Ahmet Bicer

**Affiliations:** 1 Department of Anatomy, Faculty of Medicine, Istanbul Health and Technology University, Istanbul, Turkey; 2 Department of Anatomy, Digital Imaging and 3D Modelling Laboratory, Faculty of Medicine, Ege University, 35100 Izmir, Turkey; 3 Faculty of Medicine, Dokuz Eylul University, Izmir, Turkey; 4 Department Plastic and Reconstructive Surgery, Faculty of Medicine, Ege University, Izmir, Turkey

**Keywords:** Thighs, Body contouring, Thigh liposuction, Cosmetic surgery, Lipodystrophy

## Abstract

**Background:**

The contour of the thigh is increasingly being recognized as crucial component of the ideal human physique, giving rise to heightened interest in attaining the perfect thigh profile. Notwithstanding, the contemporary landscape of cosmetic surgery appears to be bereft of efficient and precise objective methodologies to evaluate the outcomes of thigh contouring treatments. The present study is aimed to investigate the aesthetic appeal of varying thigh contours, employing specialized software as an indispensable instrument for quantitative and qualitative analysis.

**Methods:**

Standardized photographs of the lower body were obtained from a sample of 200 healthy volunteers. A linear analysis was conducted, examining aspects such as the vertical length and transvers width of the thigh, as well as angular measurements including the posterior gluteal angle (PGA) and lateral angle thigh (LAT). Variables relating to thigh measurements and body mass index (BMI) were documented, with the relationships between them ascertained through Pearson’s correlation and regression analysis.

**Results:**

In males, the LAT was measured at 168 ± 3.9, and the PGA at 170 ± 3.4, while in females, these measurements were 166 ± 2.8 ve 166 ± 2.8, respectively. Linear analyses, including the vertical length of thigh (VLT), transverse width of thigh (TWT), lateral width (LW), and posterior width (PW), were conducted. Based on the LW inferior/LW superior ratio values, the most commonly observed thigh types were Type III (0.90) at 45% and Type II (0.85) at 24.75% while the least common was Type V at 4% (0.99). PW inferior/PW superio*r* was 84.7%. The PWI/PWS ratio was highest for Type V, at 0.99, accounting for 84.70% of the total. Furthermore, an increase in the LWI/LWS ratio leads to an increase in the PWI/PWS ratio.  The frequency of the VLT/TW1 ratio 0.31-0.35 (Type 3) was found to be on the left side and Type 4 on the right side. A strong correlation was found between BMI and all thigh indexes, with a significant positive correlation between the index and factors tied to the buttocks and upper thigh.

**Conclusions:**

The concept of an ideal thigh may vary based on an individual’s gender, race, country of residence, and self-esteem, aiming to achieve a more natural silhouette. Focusing on the different ratios of hip and thigh varieties in the study is quite intriguing. Further inquiry and rigorous exploration are warranted to delineate the optimal techniques and methodologies for attaining ideal thigh proportions.

**Level of Evidence IV:**

This journal requires that authors assign a level of evidence to each article. For a full description of these Evidence-Based Medicine ratings, please refer to the Table of Contents or the online Instructions to Authors www.springer.com/00266.

## Introduction

**Fig. 1:**
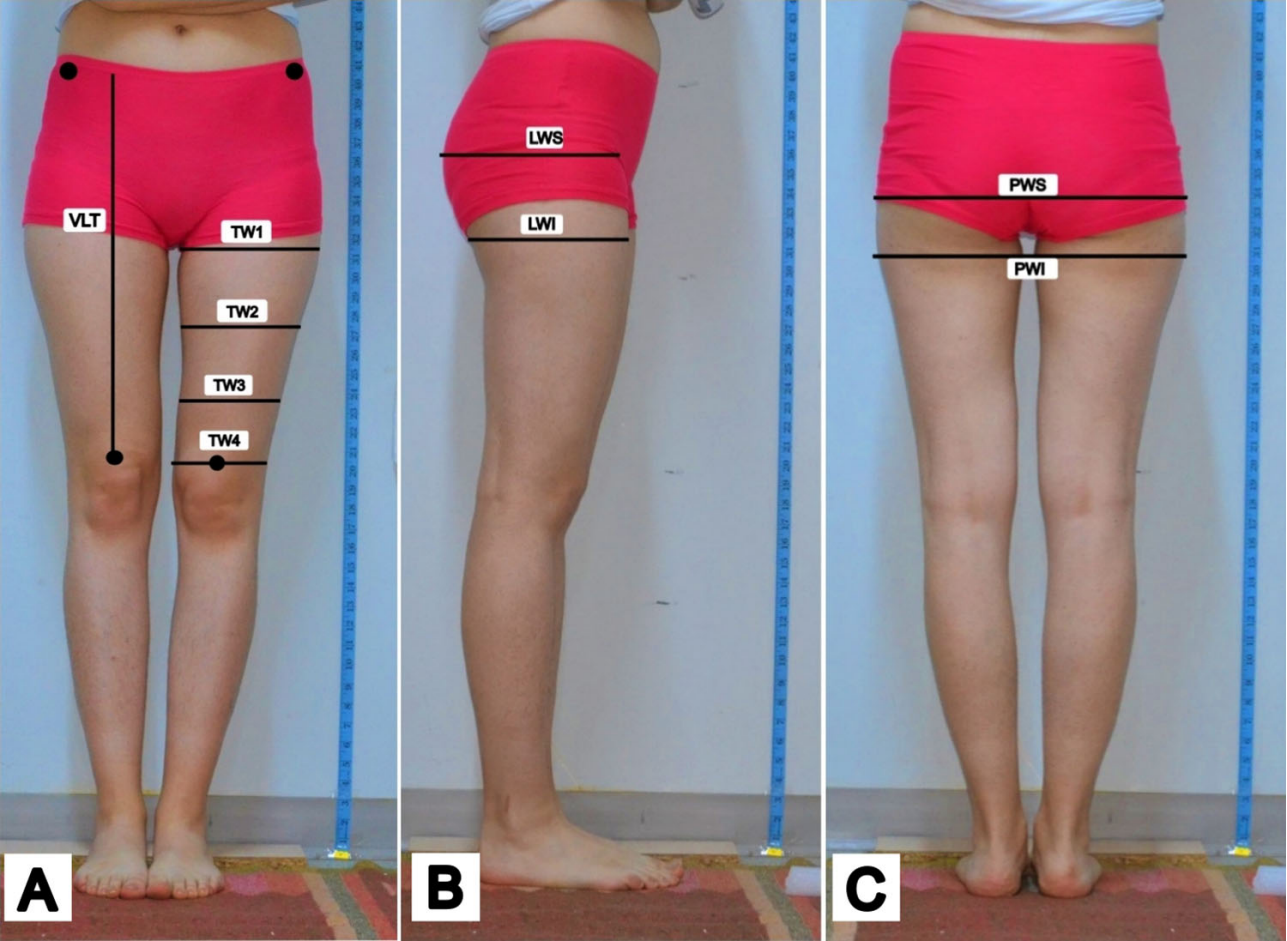
Thigh parameters. **A** *Anterior view:* vertical length of thigh (VLT), upper transverse width of thigh (TW1), 1/3 upper transverse width of thigh (TW2), 1/3 lower transverse width of thigh (TW3), and lower transverse width of thigh (TW4). **B** *Lateral view:* lateral width inferior (LWI) is the horizontal distance across the buttock–thigh junction, and lateral width superior (LWS) is the horizontal distance to the point of maximal buttock projection. **C** *Posterior view:* PWS: posterior width superior; PWI: posterior width inferior

As interest in the contouring of the lower limb region escalates within the domain of cosmetic procedures, the thigh has emerged as a salient aspect of the ideal body physique [1–3] (Fig. 1). The configuration, silhouette, and dimensions of the thigh and gluteal areas now play a pivotal role in societal perceptions of attractiveness, aesthetic appeal, and sensuality [4, 5]. Media-driven trends, coupled with the proliferation of diverse body contouring options, have precipitated a marked increase in individuals pursuing so-called perfection [6, 7]. While the body contouring methods employed to address leg contour issues are well established, certain limitations persist that complicate the practice [8–10]. First, during the consultation phase, patients often seek comprehensive solutions encompassing both medical and surgical interventions for body contouring, rather than focusing solely on localized issues. Second, the demanding pace of modern life can make it challenging for patients to take the necessary time off work for recovery. This trend is particularly evident in procedures such as liposuction, leg sculpture, selective neurectomy, micro-fat transplantation, and laser or ultrasound-assisted lipoplasty [7, 11–14].

**Fig. 2:**
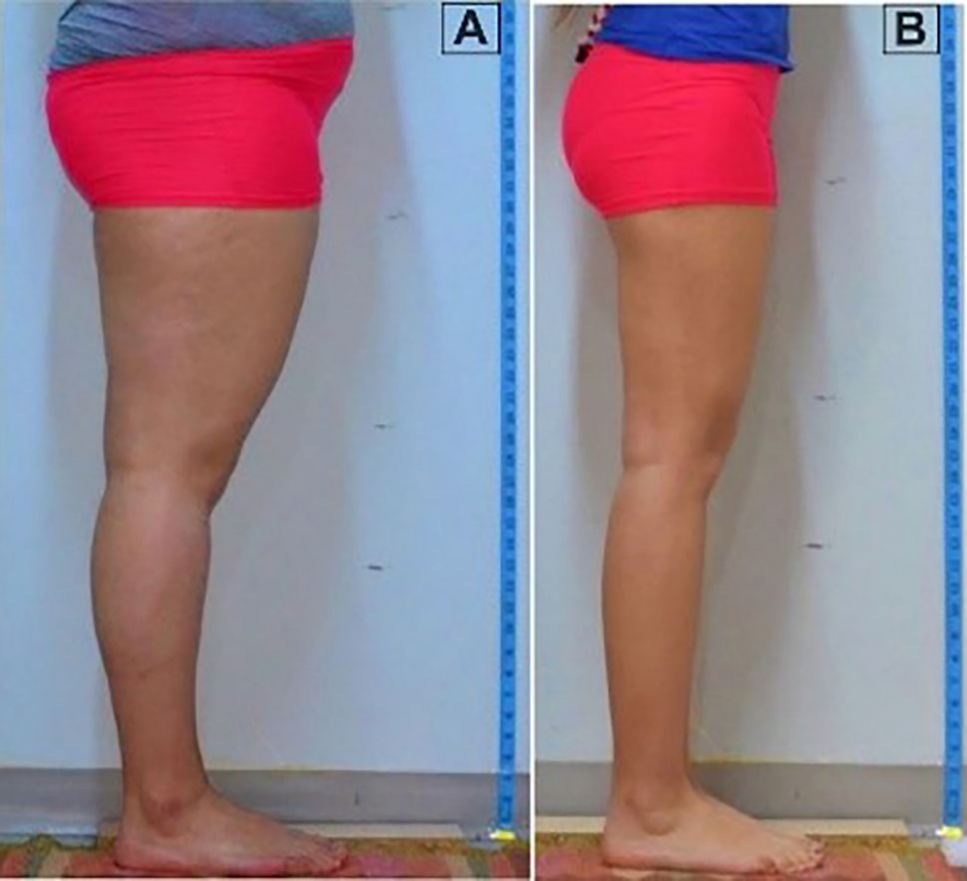
The difference in thigh profile, aesthetics, and attractiveness created by different body mass indices (**A**: 28 and **B**: 18.7) in individuals of the same age

Beginning at puberty, women often experience fat accumulation in the gynoid fat regions, specifically the thighs, buttocks, and hips [11, 16]. Roughly 85% of post-pubertal women are affected by gynoid lipodystrophy and edematous fibrosclerotic panniculopathy, commonly referred to as cellulite (Fig. 2) [8, 9]. This condition represents one of the most frequent topographical changes to the skin’s surface, particularly in the posterior-lateral thighs of post-pubertal women [1, 17]. Clinical manifestations vary widely, ranging from an “orange-peel” appearance to a “mattress-like” texture. When it comes to the consistency and proportions of the thigh region relative to the waist or hips, complaints can include an enlarged or deficient thigh, skin laxity, cellulite, or a low infragluteal fold [3, 11, 18]. Furthermore, some individuals experience overlapping skin on the inner thigh when standing, skin lesions from friction when walking, limited wide-angle thigh movement, or clothing fit issues due to a log-like thigh structure. This can lead to what some cosmetic surgeons colloquially refer to as saddlebag deformity or big squish marshmallow on a stick. The resulting figure may be perceived as unattractive and, thus, counterproductive to patients’ desires. These issues often have detrimental effects on quality of life. In both genders, a sedentary lifestyle, the obligation to stay at home created by the pandemic, and spending excessive time with computers and TVs have caused similar thigh problems not only in women but also in men, leading to thigh contour problems in both sexes [6, 10, 19–21].

In the investigation of the highly popular subject of the ideal thigh, there exist a surprisingly limited number of recent studies that describe the anatomical criteria of the thigh region in terms of shape, contour, and dimensions [2, 4, 8, 9, 15, 18, 22–24]. Much of the existing research has concentrated on three key determinants of thigh beauty lateral width inferior (LWI)/lateral width superior (LWS), body mass index (BMI), and posterior width inferior (PWI)/posterior width superior (PWS) in both sex [1, 5, 9, 17]. Although these factors have been studied, a comprehensive analysis is needed to ascertain whether thigh measurements and profiles align with what is considered attractive and ideal [5, 7, 9, 11, 20]. As the demand for aesthetic thigh liposuction continues to surge, a renewed focus on defining the ideal thigh contouring has emerged. Nevertheless, there is a significant gap in the literature that characterizes an ideal thigh, despite its substantial influence on the overall gluteal aesthetic. While numerous studies have analyzed the objectives and outcomes in gluteal augmentation, there is an absence of the literature discussing the specific role of the thigh contour in overall aesthetics lower limb [13, 25].

Quantitative methodologies such as digitalized photogrammetry and three-dimensional imaging have been established as invaluable and pragmatic tools for evaluating ideal thigh, the aesthetic profile of leg beauty, and the smoothness and symmetry of the thighs [26, 27]. Procedures aimed at volume restoration and contouring symmetrically emphasize the thighs and buttocks, thereby aiding and in the achievement of optimal lower limb projection [4, 5, 20, 24]. Such an approach facilitates the creation of a more natural curvature, yielding an aesthetically satisfying result for both the thigh and the neighboring buttocks area following any aesthetic intervention. Consequently, it becomes imperative to enhance our comprehension of the entire leg sculpture, including the interrelationship between leg variables and BMI. Undertaking meticulous measurements of the lower limb is pivotal in devising a personalized treatment plan for each patient. The objective of this study is to scrutinize the thigh region using reference measurements, thereby contributing to the understanding of what constitutes the ideal thigh.

## Materials and Methods

### Patient Demographics

**Fig. 3:**
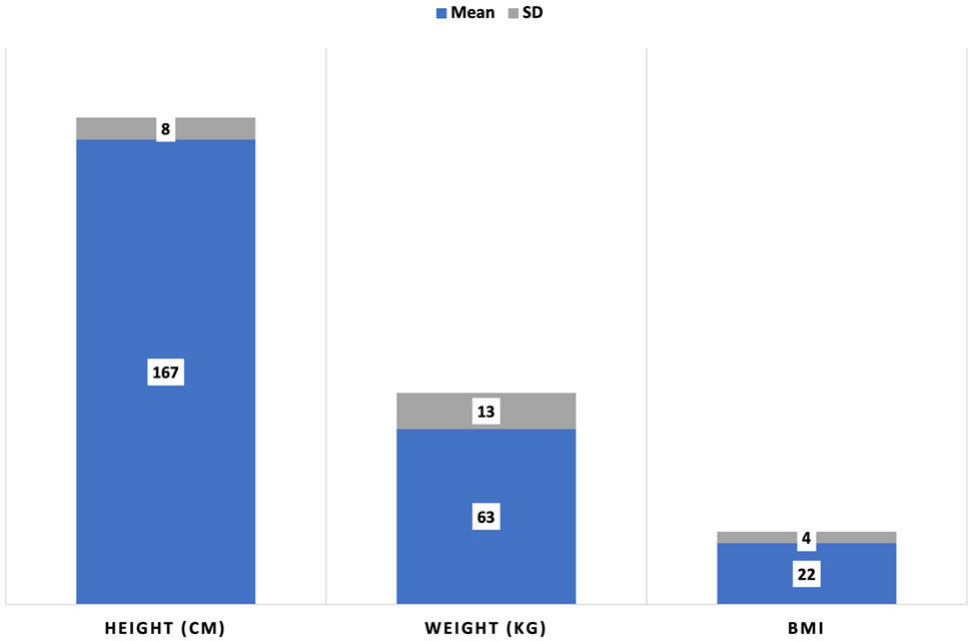
The height, weight, and body mass index (BMI) values of the volunteers participating in the study

**Fig. 4:**
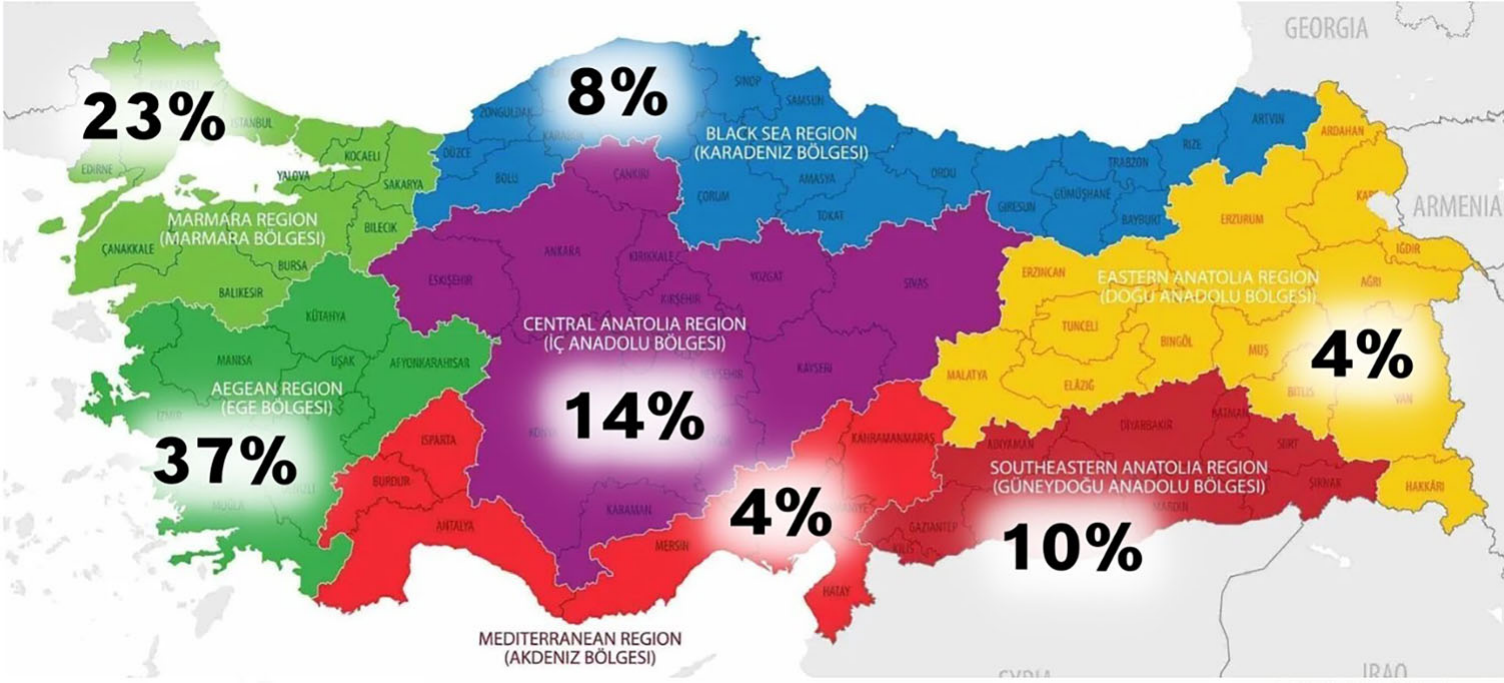
The distribution of participants according to regions of Turkey (Marmara region, Black Sea of Region, Aegean Region, Mediterranean Region, Central Anatolia Region, Eastern Anatolia Region, Southeastern Anatolia)

This study included 200 volunteer adults (100 men, 100 women) aged 19 to 21 years, all without lower body anomalies (Fig. 3). The exclusion criteria encompassed obesity (BMI > 30), underweight (BMI < 18.5), any history of congenital hip dislocation, significant trauma or operations in the lower limb regions, and pregnancy. The participants can be described as people living in Anatolia. In terms of race, it could be said they are of Caucasian and Middle Eastern mixed race. In our study, the participants were questioned about their place of birth according to the regions of Turkey (Marmara region, Black Sea of Region, Aegean Region, Mediterranean Region, Central Anatolia Region, Eastern Anatolia Region, Southeastern Anatolia) (Fig. 4), their sports activity habits (whether they practiced sports for at least 6 months or more), and the type of sports they were interested in. In addition, the frequency of vegetable consumption and white meat consumption among dietary habits was questioned with the options “Once or more a day; 4 to 6 times a week; 1 to 3 times a week; Less than once a week; Never.” The research received approval from the duly established Ethical Committee at Ege University’s Research division and adhered to the standards set forth in the Declaration of Helsinki (approval number: 16-10.1/14).

### Digital Photogrammetry

**Fig. 5:**
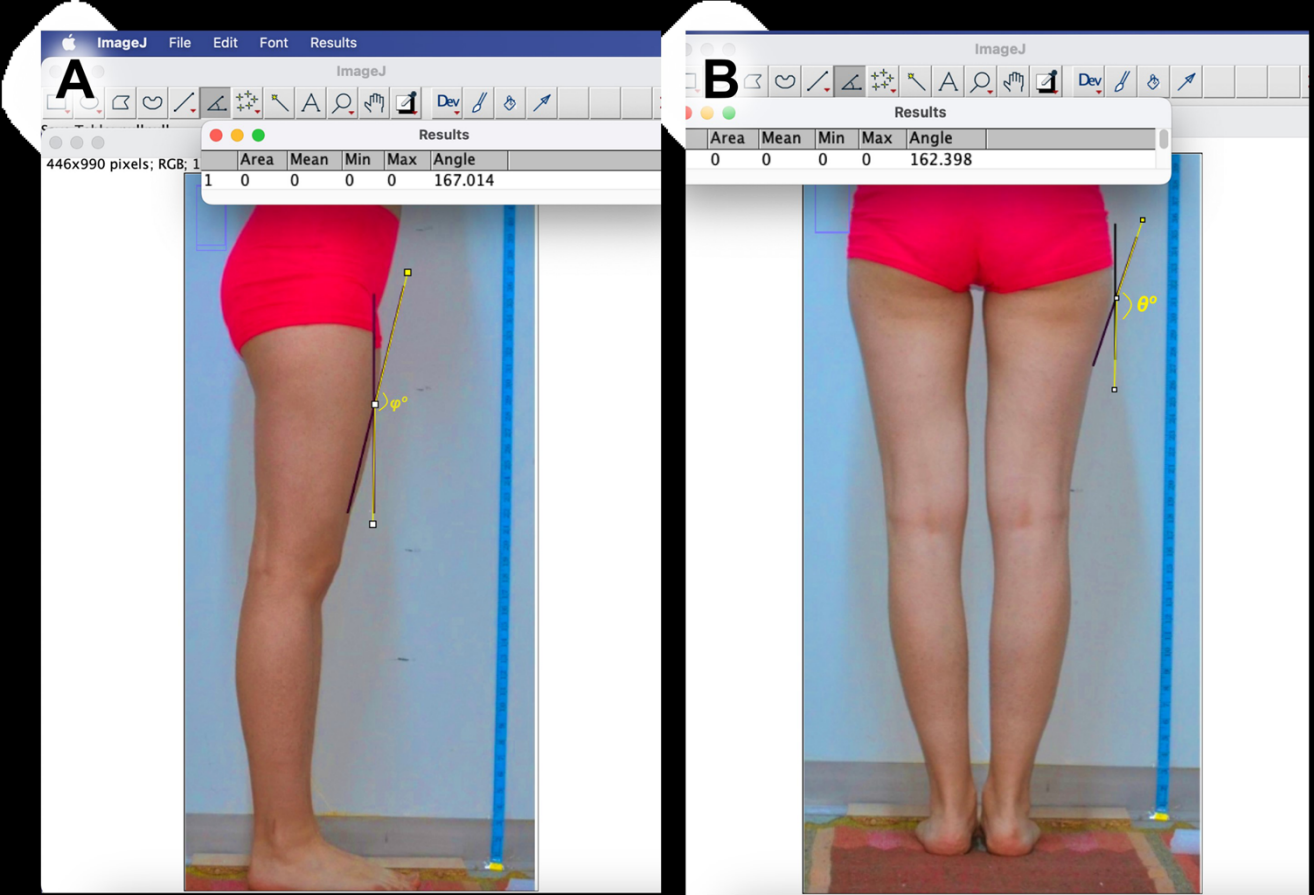
**A** Measurement as lateral angle of the thigh (*φ*) was conducted using the lateral view, employing Image J software version 1.47 for the analysis. **B** In the posterior view, the posterior gluteal angle (*θ*). This angle is formed between two lines: The first line is an anatomical vertical meridian that extends from the anterior superior iliac spine to the trochanteric crest, which is transposed laterally in the diagram to intersect the thigh–buttock convexity, and the second line is an oblique line that runs from the widest point of buttock projection to the thigh–buttock junction

**Fig. 6:**
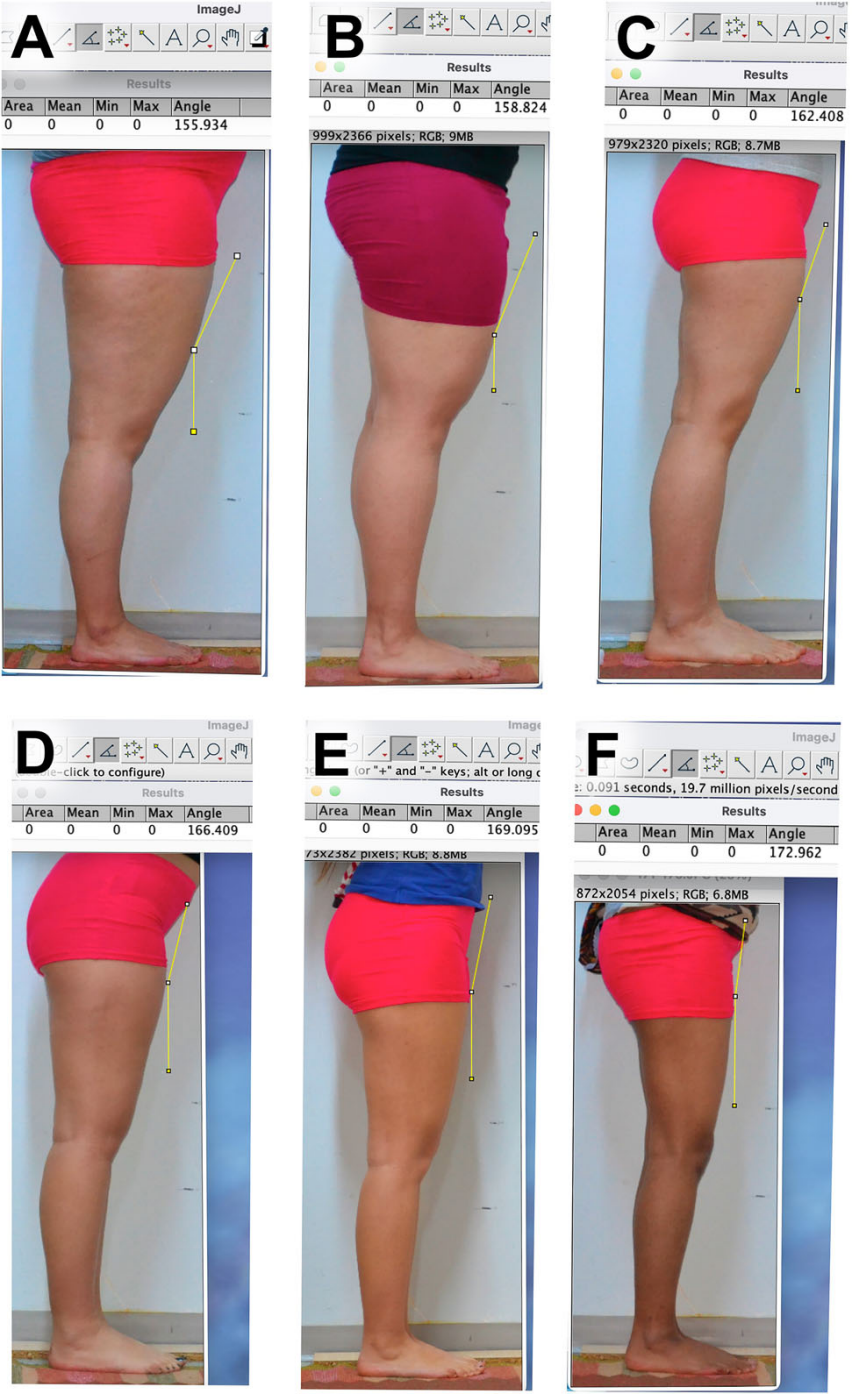
Lateral view showing variations in lateral angle thigh in women as **A**: 154-156°, **B**: 157-160°, **C**: 161-164°, **D**: 165-168°, **E**: 169-171°, **F**: 171-175°

The anatomy of the thigh region was evaluated through digital images captured from standard photographs of the subjects’ lower bodies (Fig. 1). These images were taken from anterior (Fig. 1A), lateral (Figs. 1B and 5A), and dorsal (Figs. 1C and 5B) views with the subjects standing upright and feet together. The distance from the object to the lens was set at 100 cm, and priority mode (GC) was employed for the lens. The captured images were subsequently transferred to a personal computer, where Image J 1.48v software facilitated the calculation of distances, angles, and ratios (Figs. 5, 6). All the landmarks used for the lower limb assessments were identified, as depicted in Figs. 5, 6 and Tables 1-3. These anatomical landmarks served as reference points in the thigh region. Linear analyses such as vertical length of thigh (VLT), transvers width of thigh (TWT), lateral width superior (LWS), lateral width inferior (LWI), posterior width superior (PWS), and posterior width inferior (PWI) were conducted. Angle measurements such as posterior gluteal angle (PGA) and lateral angle of thigh (LAT) were calculated and classified according to these reference points (Fig. 5). In anterior view, vertical length of thigh/upper transvers width of thigh (VLT/TW1), vertical length of thigh/1/3 upper transvers width of thigh (VLT/TW2), vertical length of thigh/1/3 lower transvers width of thigh (VLT/TW3), vertical length of thigh/lower transvers width of thigh (VLT/TW4) were calculated (Fig. 1, Table 2).

**Table 1:** Measurement of reference angles in the thigh region

Features	Measurements		
	Lateral angle of thıgh	Posterıor gluteal angle	*p*
Right	165 ± 3.8 (154–179)	167 ± 4.2 (157–179)	*p* > 0.05
Left	167 ± 2.8 (155–178)	167 ± 3.8 (154–177)	
Male	168 ± 3.9 (156–179)	170 ± 3.4 (164–174)	*p* > 0.05
Female	166 ± 2.8 (154–174)	166 ± 2.8 (154–174)	
Total	166 ± 2.8 (154–174)	165 ± 3.8 (154–179)

**Table 2:** Classification of nine distinct thigh types based on the linear measurement ratios in the thigh region

Sıde		Thıgh classıfıcatıon									
		Type 1 0.21-0.25	Type 2 0.26-0.3	Type 3 0.31-0.35	Type 4 0.36-0.4	Type 5 0.4-0.45	Type 6 0.46-0.5	Type 7 0.51-0.55	Type 8 0.56-0.6	Type 9 0.61-0.65	*P*
Rıght	VLT/TW1	0	0	0	4.35	25.45	40.35	23	5.6	1.25	>0.05
	VLT/TW2	0	4.35	31.05	**39.75**	19.25	4.35	1.25	0	0	>0.05
	VLT/TW3	3.7	23.6	**48.45**	19.25	4.35	0.65	0	0	0	>0.05
	VLT/TW4	13	**44.75**	37.9	3.1	1.25	0	0	0	0	>0.05
Left	VLT/TW1	0	0	4.94	29.2	37.9	21.1	6.21	0.65	0	>0.05
	VLT/TW2	0	4.95	24.85	**41.6**	23.6	4.35	0.65	0	0	>0.05
	VLT/TW3	3.7	27.95	**40.35**	24.85	2.5	0.65	0	0	0	>0.05
	VLT/TW4	14.3	**48.45**	33.55	2.4	0.65	0.65	0	0	0	>0.05

VLT/TW1: Vertical length of thigh/upper transvers width of thigh, VLT/TW2: Vertical length of thigh/1/3 upper transvers width of thigh, VLT/TW3: Vertical length of thigh/1/3 lower transvers width of thigh, VLT/TW4: Vertical length of thigh/lower transvers width of thigh.

### Thigh Shape

**Fig. 7:**
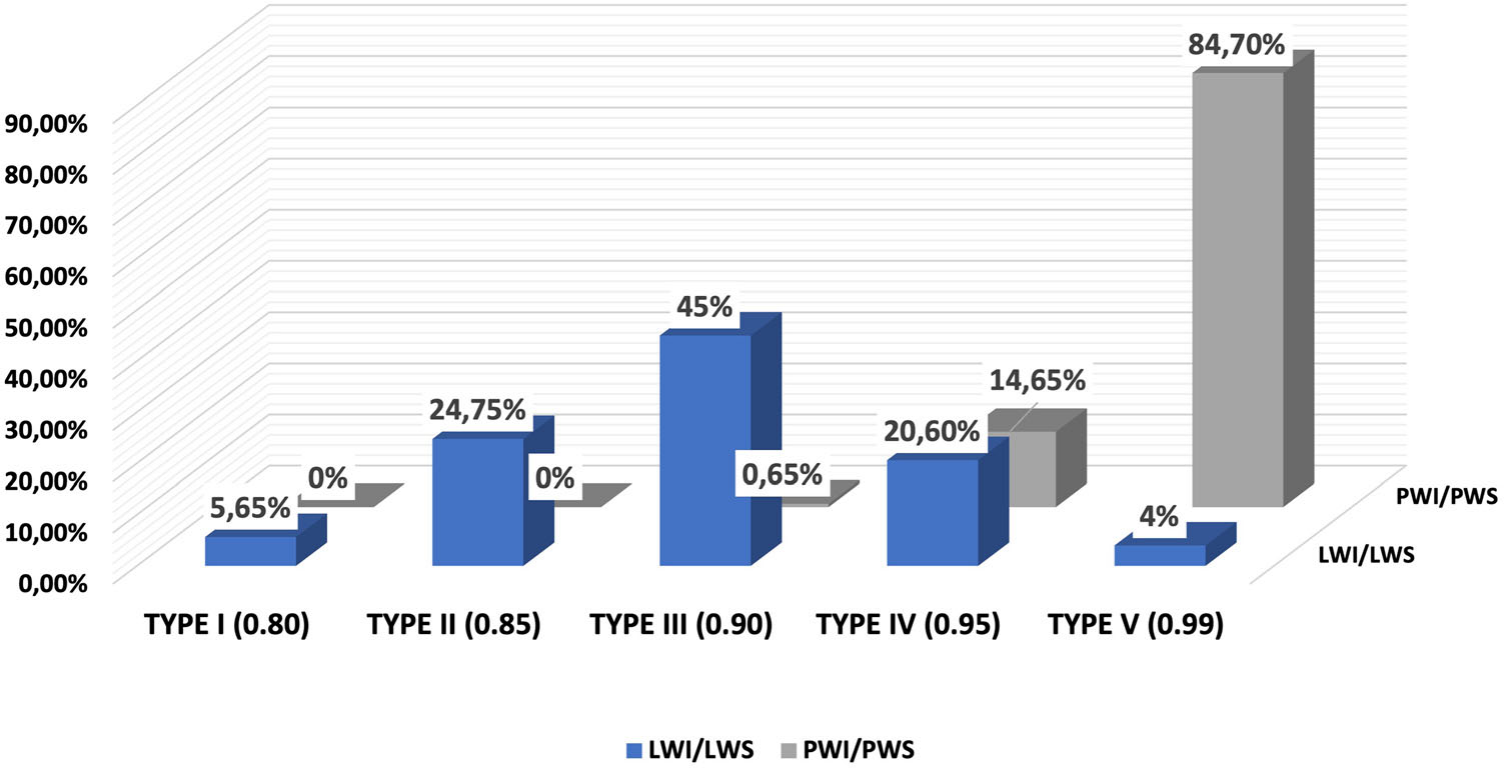
Frequency of linear analysis ratio across diverse thigh types. LWI/LWS: Lateral width inferior/Lateral width superior, PWI/PWS: Posterior width inferior/Posterior width superior

In anterior view, thigh typing was organized into nine distinct categories based on the values of the VLT/TW1, VLT/TW2, VLT/TW3, and VLT/TW4 ratios, utilized 5% increments (Table 2). The thigh was classified into nine groups as Type 1 (0.25), Type 2 (0.3), Type 3 (0.35), Type 4 (0.4), Type 5 (0.45), Type 6 (0.5), Type 7 (0.55), Type 8 (0.6), and Type 9 (0.65) (Fig. 6). In both the posterior and lateral views, the thigh profile was categorized into five types: Type I with a ratio of 0.80, Type II with a ratio of 0.85, Type III with a ratio 0.90, Type IV with a ratio of 0.95, and Type V with a ratio of 0.99 (Table 3 and Fig. 7).

**Table 3:** Classification of thigh types based on the measurement ratio of the posterior and lateral views in the thigh region

Ratıo	Thıgh classıfıcatıon					
	Type I 0.75–0.80	Type ıı 0.81–0.85	Type III 0.86–0.90	Type IV 0.91–0.95	Type V 0.96–1.00	Total
LWI/LWS	5.65%	24.75%	**45**%	20.6%	4%	100
PWI/PWS	0	0	0.65%	14.65%	**84.7**%	100

LWI/LWS: Lateral width inferior/Lateral width superior, PWI/PWS: Posterior width inferior/Posterior width superior

### Statistical Analysis

All the data were processed through a correlation matrix using the SPSS 7.5 software program (Upper Saddle River, N.J.: Prentice Hall, © 1996). A statistical significance level was set at *p* < 0.05. The relationships among thigh measurements, ratio, and body mass index were examined using Pearson’s correlation analysis method. Furthermore, regression analysis was conducted to explore the association between all quantitative variables and the body mass index. The levels of statistical significance were as *p* < 0.05 and *p* < 0.01.

## Results

### Subject

**Fig. 8:**
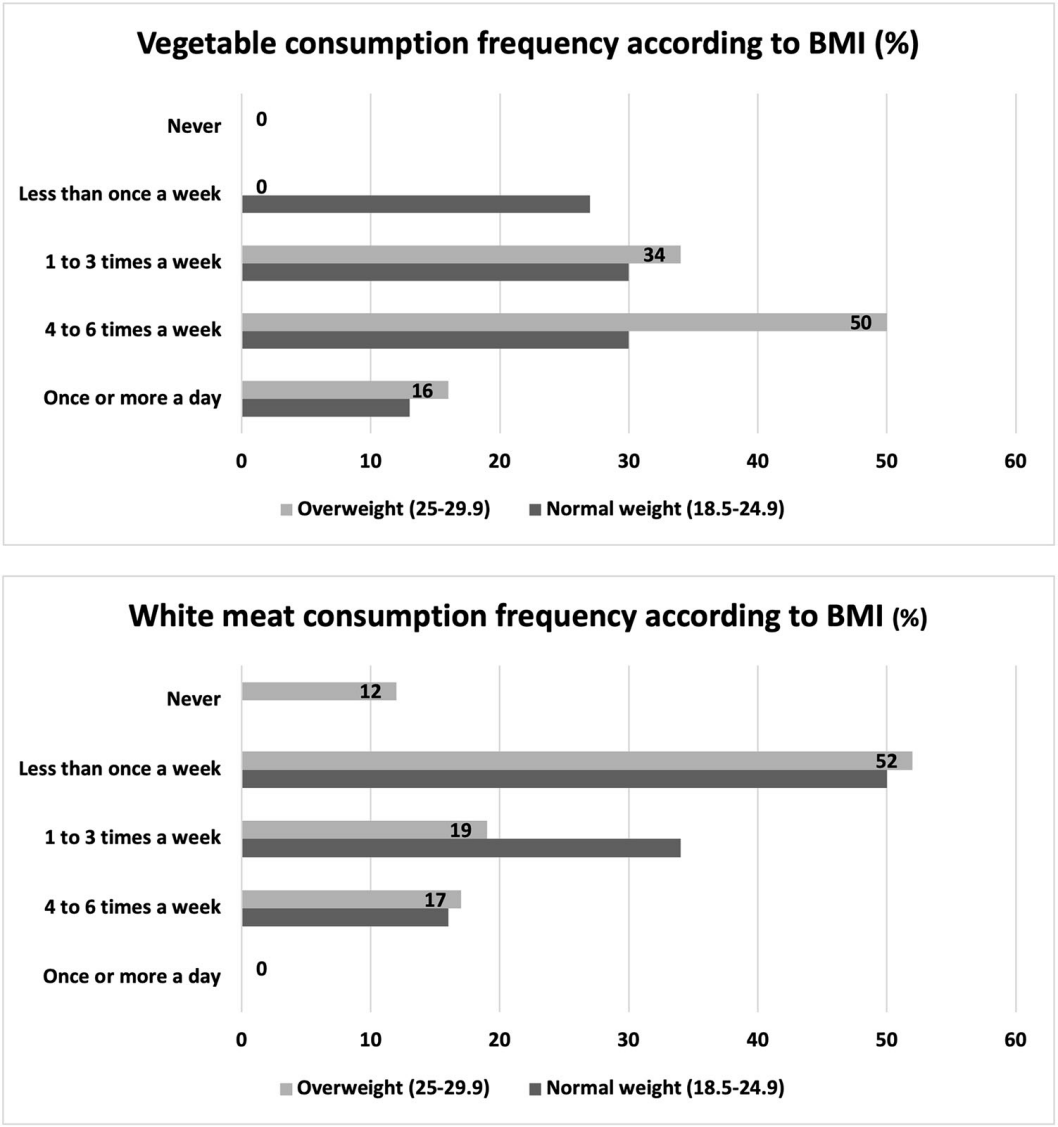
The findings obtained regarding the participants’ vegetable and white meat consumption habits and their relationship with BMI

The BMI was classified as BMI 18.5-24.99 in 79%, BMI 25-29.99 in 17%, BMI 30-39.99 in 4% frequency, and BMI > 40 in 0%. Half (50%) of the participants in the overweight group consumed vegetables 4-6 times a week, while 52% consumed white meat less than once a week. Vegetable consumption of participants with normal BMI was found that 60 participants consumed vegetables 4-6 times a week and 60 participants consumed vegetables 1-3 times a week (Fig. 8). According to the answers obtained from the survey questions, 45% of the participants were regularly involved in sports (fitness and football) for at least 6 months or more.

### Thigh Measurements

**Fig. 9:**
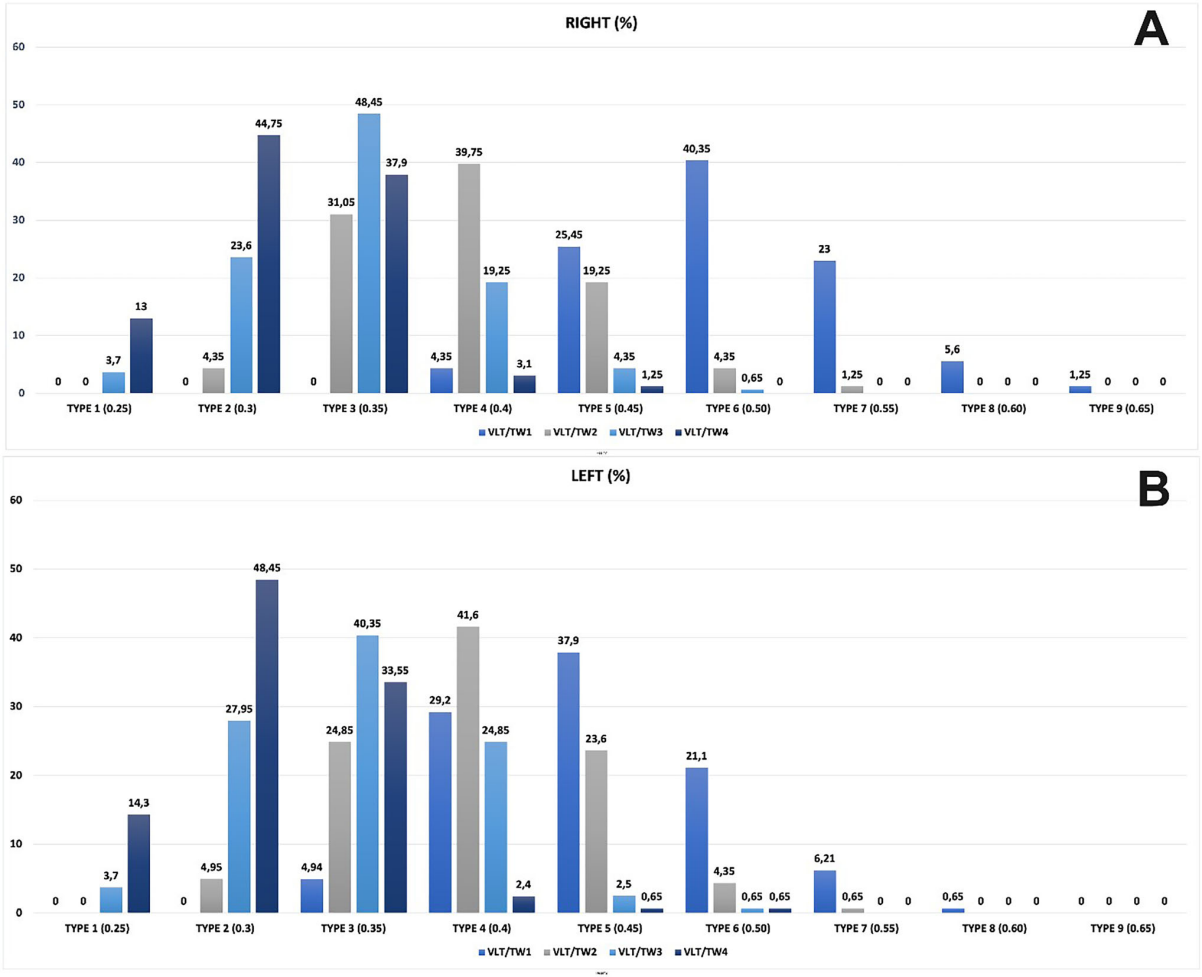
Differences in ratios right and left legs in various thigh types, as represented by frequency. VLT/TW1: Vertical length of thigh/upper transvers width of thigh, VLT/TW2: Vertical length of thigh/1/3 upper transvers width of thigh, VLT/TW3: Vertical length of thigh 1/3 lower transvers width of thigh, VLT/TW4: Vertical length of thigh/lower transvers width of thigh

The quantitative analysis of anterior, lateral, and posterior views of thighs, using designated reference points, is delineated in Tables 1-3 and Figs. 7 and 9.

The VTL/TW1, VTL/TW2, VTL/TW3, and VTL/TW4 values were computed. For all ratios, the most common type of Type 3 was on the right side and Type 2 on the left side. The most effective types have been identified as Types 2, 3, and 4 (Table 2). Regarding the VLT/TW2 ratio, Type 4 was the most common on both sides. According to the results of the VLT/TW3 ratio, Type 3 was the most prevalent. Finally, when examining VLT/TW4, Type 2 was found to be the most observed type on both sides (Table 2 and Figs. 7 and 9).

Regarding the thighs, based on the VLT/TW1, VLT/TW2, VLT/TW3, VLT/TW4 values, it can be stated that the right and left thighs are asymmetrical for Types 3, 4, 5, 6, 7, and 8. The difference may be associated with the dominant extremity.

Based on the LWI/LWS ratio values, the most commonly observed thigh types were Type III (0.90) at 45% and Type II (0.85) at 24.75%, while the least common was Type V at 4% (0.99). For the PWI/PWS ratio, the most common was Type V (0.96-0.99) accounting for 84.7%. Therefore, we observe that as the LWI/LWS ratio increases, the PWI/PWS ratio also increases (Fig. 7). According to our findings and graphs, a decrease has been observed in both minimum and maximum measurements of the thigh as one moves from the upper to the lower part of the limb (Table 2 and Fig. 6). The statistical significance of this decrease is calculated to be 0.05, indicating that the reduction was systematic. No instances of a uniformly thick or log-like thigh profile were encountered.

According to the answers obtained from the questionnaire questions about the sports activity of the volunteer participants, it was determined that 90 individuals of them had been doing sports regularly for at least 6 months or more. The ratios of our morphometric measurements (VTL/TW1, VTL/TW2, VTL/TW3, and VTL/TW4) and angle measurements (lateral angle of thigh and posterior gluteal angle) were statistically compared between the participants who practiced sports and those who did not. According to the findings obtained, a significant difference was seen between the “VLT/TW1 (*p* < 0.01; *r* = 0.584) and VLT/TW2 (*p* < 0.01; *r* = 0.719)” parameters among the parameters.

## Discussion

Well-projected thighs, extending in a smooth continuum from the waist to the knee on the frontal view, create a natural and aesthetically pleasing curve (Figs. 1, 2) [1–4, 6, 7, 18]. Although both female and male icons in various studies are often represented as the epitome of beauty, they exhibit unique characteristics and may not necessarily embody the average body type or aesthetic ideals embraced by the broader population [7, 9, 22]. In the pursuit of defining the ideal buttocks, previous studies have acknowledged the complex interplay among various factors such as region, sex, age, occupation, and specific anatomical details. This has led to a commonly recognized waist-to-hip ratio that seems to resonate across diverse cultures and geographical locales [2, 15, 17, 20, 28, 29]. Yet, a definitive standard for the ideal thigh remains elusive.

The aesthetic perception of the thigh region’s shape is an integral component of overall body and leg profiles, with procedures such as liposuction, fat grafting, and related interventions like aspiration of oil cyst and graft harvest witnessing a rapid surge in popularity [11, 12, 14, 21, 25, 28, 30]. This competition has precipitated a demand for innovative, safe, effective, and flexible surgical techniques, treatment algorithms, and strategies. From a surgical perspective, optimal sculpting of the thigh region can be achieved by tailoring solutions to individual anatomy and adhering to precision based on mathematical metrics.

**Fig. 10:**
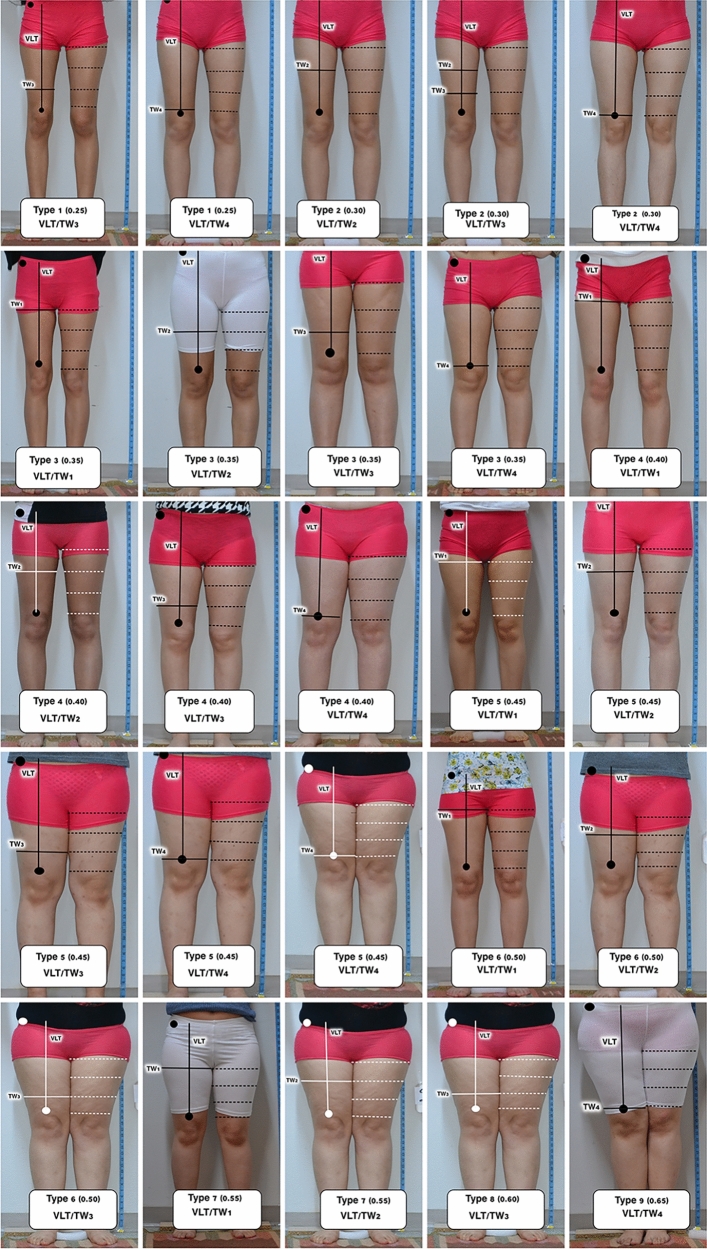
Demonstration of different types according to morphometric variations of the thigh

In this study, we have presented the morphological attributes of the thigh region by employing digitalized standard lower body photographs from healthy subjects and further delineated gender-related distinctions utilizing computer-aided technology (Figs. 1, 2, 5, 6, 8–10). Photogrammetry stands as a prevalent method for noninvasive evaluation of body segments, offering an advantage over radiographic approaches by negating radiation exposure and obviating the need for printing. The aim of this research was to furnish reference metrics for analyzing the thigh in both sagittal and coronal planes, anterior, lateral, and posterior profiles, employing computerized photogrammetry in a population of healthy young adults. Our results imply a preference for additional breadth in the thigh’s coronal plane over the sagittal plane. Over-extension of thigh width in a side view yielded unpleasing results, while a range of thinner thighs was considered equally acceptable. These insights align with contemporary preferences for a curvy and feminine silhouette. Augmenting the lateral aspects of the thighs, particularly adjacent to the gluteal crease, fosters a more fluid transition from the buttock to the leg, thereby avoiding an unnatural mismatch.

Our findings suggest that young individuals present a BMI ranging from 18.14 to 22.49. However, since the BMI serves as a general gauge for body contour assessment, localized evaluations of distinct body regions would yield more nuanced insights. Accordingly, it is imperative to probe the interaction between general and specific parameters by addressing the main question: What correlations exist between thigh variables and BMI? Pearson’s correlation analysis revealed a substantial correlation between BMI and thigh ratios (*p* < 0.01), with the strongest positive association with LAT and PGT (Pearson correlation coefficient, 0.682). This implies that a higher BMI is linked with greater upper thigh measurements (Fig. 2). A linear relationship was also identified between the increase in BMI and the narrowing of the angles (LAT and PGT), and an increase in thigh ratios was observed.

Total leg sculpture offers an amalgamated aesthetic solution for enhancing limb shapes, with higher BMI values corresponding to better aesthetic improvements. The study details the outcomes of total leg sculpture and examines the significance and correlations among thigh pattern variables, gender, and BMI through statistical scrutiny. Data including thigh pattern variables, gender, BMI, and transverse change rates across three views and two planes were evaluated.

The study presents a detailed quantitative analysis of thigh measurements, classifying them into nine types. Various ratios were computed including LWI/LWS, PWI/PWS, VTL/TW1, VTL/TW2, VTL/TW3, and VTL/TW4. The VLT/TW1 ratio should not be high, and Types 1 and 9 are considered non-preferred categories for aesthetic purposes. Surgical intervention could be considered for these individuals. From the perspective of the anterior thigh profile, the VLT/TW3 and VTL/TW4 ratios should fall within the range of 0.3–0.4. In the lateral profile, the LWI/LWS ratio should ideally be between 0.86 and 0.90, and the PWI/PWS ratio should be between 0.96 and 1.00 for preferred aesthetic outcomes. The findings revealed specific trends and commonalities among these ratios, such as the most and least common types for LWI/LWS and PWI/PWS ratios. There was also an observation that as the LWI/LWS ratio increased, the PWI/PWS ratio similarly increased. Additionally, the analysis demonstrated asymmetry between the right and left thighs for Types 3 to 8, while symmetry was noted for Types 1, 2, and 3 within the sample group. Figures 5 and 6 provide visual representations of these findings.

The volunteers measured in our study were young adults. It has been chosen from people who have not given birth before, are old enough to devote more time to sports, and have not started their business life. It was studied with these volunteers that the thigh measurements did not change and were more ideal. It was determined that the most common thigh types in our groups were actually beauty types.

The limitations of the study are defined as young adults who have not given birth and have no disabilities preventing them from engaging in physical activity. The study provides and discusses details and parameter changes related to the volunteers’ participation in sports. Beyond this, the variety of sports engaged in by the volunteers could have been detailed further. For example, differences between individuals who participate in swimming or athletics could have been explored. A distinction in this regard was not made. This can be targeted with a comprehensive study with wider participation.

Another limitation is that the significance of anatomical and anthropometric findings for the male population is currently quite limited. However, demand for these findings may increase in the context of gender transition.

This study finds the focus on different ratios of hip and thigh varieties quite intriguing in achieving the desired outcomes (Figs. 2, 6, 7, 9, 10). While the study’s findings on anthropometric measurements and the classifications made with them offer very interesting data on their own, they may not be directly linked to surgical indications. We are currently avoiding drawing any conclusions on aesthetic issues based on our study findings. This is because the necessity for correction in thigh shape can vary depending on an individual’s gender, race, the country they live in, self-esteem, etc.

What is considered “beautiful” this year could be undesirable 10 years from now. This situation is particularly relevant today in the context of feminine/masculine distinctions. In the past, certain decades have made the masculine appearance in women fashionable, while the next could do the complete opposite.

## Conclusion

The integration of digital technology into pre-surgical decision-making enables surgeons to anticipate treatment outcomes, enhance risk management, and garner more personalized information for patients. Advanced topographical technology elevates the standards of planning in resurfacing procedures. This investigation is focusing on the different ratios of hip and thigh varieties in the study is quite intriguing.
